# High Adhesion of Tumor Cells to Mesothelial Monolayers Derived from Peritoneal Wash of Disseminated Gastrointestinal Cancers

**DOI:** 10.1371/journal.pone.0057659

**Published:** 2013-02-25

**Authors:** Danilo Ranieri, Salvatore Raffa, Andrea Parente, Simone Rossi Del Monte, Vincenzo Ziparo, Maria Rosaria Torrisi

**Affiliations:** 1 Institute Pasteur-Fondazione Cenci Bolognetti, Department of Clinical and Molecular Medicine, Sapienza University of Roma, Roma, Italy; 2 Department of General Surgery, Sapienza University of Roma, Roma, Italy; 3 Sant’Andrea Hospital, Roma, Italy; University Magna Graecia, Italy

## Abstract

The role of the mesothelial layer in the peritoneal spreading of cancer cells is only partially clarified. Here we attempted to better define the mesothelial contribution to the tumor cell adhesion using a direct adhesion test applied to human primary cultures of mesothelial cells (HPMCs) derived from the peritoneal washes of patients with gastric and colorectal cancers. Gastric and colon carcinoma cells were seeded on different mesothelial monolayers and quantitative fluorescence analysis was performed to analyze their growth and adhesive properties. The adhesion of the cancer cells was not affected by the origin of the HPMCs when derived from patients with different cancers or with benign disease. In contrast, the high levels of ICAM1 expression and ROS production, which characterize these senescent mesothelial cells, enhanced the tumor cell adhesion. These results suggest that the mesothelial adhesive properties are dependent on the cell senescence, while are not affected by the tumor environment. The use of peritoneal washes as a source to isolate HPMCs provides a practical and reliable tool for the in vitro analysis of the mesothelial conditions affecting the peritoneal carcinomatosis.

## Introduction

The peritoneal spreading of gastric and colorectal cancers represents a frequent event occurring after curative resection [1]–[3]. Critical for the peritoneal recurrence is the adhesion of the free disseminated cancer cells to the mesothelial layer and many different molecular mechanisms directly involved in this process have been identified [4]. For peritoneal carcinomatosis, cancer cells must be able to survive in the peritoneal cavity, once detached from the primary tumor, and must display a proliferative and invasive behaviour, once adhered to the mesothelium. While many studies have been addressed to the analysis of the expression and activation of molecular pathways responsible for the sequential biological changes of the different types of cancer cells [5]–[7], only a limited number of reports have focused on the contribution of the mesothelial layer in the adhesion and peritoneal spreading of the cancer [8]–[10].

For the detailed analysis of the molecular mechanisms affecting the adhesive stage, different in vitro or ex-vivo models have been developed [11]–[13] and primary cultures of mesothelial cells have been obtained to test the adhesion of cancer cells in presence of promoting or interfering agents [8], [12]. Most of these models utilize either established cell lines or human primary cultures of mesothelial cells isolated from omental fragments [10], [14]–[15]. However it has been proposed that also the peritoneal lavages, being the gold standard for assessing the presence of peritoneal dissemination of gastric and colorectal cancer [16]–[18], are a good and more practical source of mesothelial cells to be propagated in vitro [19], although their use in co-culture models has not been explored.

Adhesion molecules play a major role in the step involving the attachment of the free cancer cells to the peritoneal surface [4] and cytokines, such as interleukin 1ß (IL1ß) and tumor necrosis factor α (TNFα) released in the inflammatory microenvironment, are known to promote their expression [20], [21]. Among the adhesion molecules which play a key role in the spreading of the neoplastic cells to the mesothelial monolayer, several studies pointed to the specific function of the intercellular adhesion molecule 1 (ICAM1) present on the mesothelial cells in promoting the process [10], [21]; in addition, it has been shown that the up-modulation of its expression, as a result of oxidative stress and senescence of the peritoneal cells, promotes the adhesion of neoplastic cells from ovarian, gastric and colon cancers [22]–[24], demonstrating the general and crucial role of ICAM1 in the spreading.

In the attempt to better define the mesothelial contribution to the adhesion of cancer cells and, in particular, the possible role of the mesothelial activation in a cancerous environment mimicking in vitro as much as possible the in vivo conditions, we used here a direct adhesion test performed on human primary cultures of mesothelial cells (HPMCs) derived from the peritoneal washes of patients with gastric and colorectal tumors or of patients with benign diseases, in order to mimic in vitro as much as possible the in vivo conditions. With the aim to minimize the possible variations attributable to the tumor counterpart, we matched different isolated HPMCs, grown also at different levels of senescence, with two well known cancer cell lines. Our results show that the adhesive behaviour of the cancer cells is not affected by the origin of the HPMCs from patients with different tumors. However, our observations confirm the role of the peritoneal senescence, through the enhanced production of reactive oxygen species and of ICAM1 expression, in promoting the tumor cell adhesion [22]–[24] and suggest that the use of the peritoneal washes as a source to isolate and propagate HPMCs can be easily applied to evaluate in vitro the state of the mesothelium in cancer patients.

## Materials and Methods

### Cell lines

The human mesothelial MeT-5A cell line [25] was cultured in Dulbecco’s Modified Eagle’s/F12 Medium (DMEM/F12) supplemented with 10% fetal bovine serum (FBS) plus antibiotics and hydrocortisone (0,1 µg/ml), insulin (2,5 µg/ml), transferrin (2,5 µg/ml) and selenium (2,5 ng/ml) (Sigma Chemicals Co., St Louis, MD, USA). The human colorectal adenocarcinoma Caco2 cell line [18], [26] was cultured in Dulbecco’s Modified Eagle’s Medium (DMEM) supplemented with 10% FBS plus antibiotics (Sigma). The human gastric adenocarcinoma AGS cell line [18] was cultured in Ham’s F12 Medium (Sigma) supplemented with 10% FBS plus antibiotics (Sigma).

### Primary cultures and co-cultures

Primary cultures of Human Peritoneal Mesothelial Cells (HPMCs) were obtained from intraoperatively peritoneal lavages [18] of patients affected by peritoneal carcinomatosis from colorectal cancer (#062) or gastric cancer (#219) as well as of patients affected by non-cancerous disease (#002), who underwent surgery at the A Unit of Surgery of Sant’Andrea Hospital. The donor clinicopathological characteristics are described in Table 1. All patients were extensively informed and gave written consent for the investigation. The protocol of the study was approved by the Ethical Board of Sant’Andrea Hospital, Rome.

**Table 1:** Clinicopathological characteristics of the peritoneal wash donors.

HPMC culture	Sex	Age	Histology	Stage	Grading	Peritoneal carcinomatosis
#002	F	42	pseudomembranous colitis	–	–	–
#062	F	70	colon adenocarcinoma	T4N1M0	G2	Yes
#219	M	75	gastric adenocarcinoma	T4N2M1	G3	Yes

To avoid possible activation of the peritoneal cells by the surgical process, the peritoneal lavages were obtained at the starting steps of the surgery. From each patient, 40 mL of peritoneal wash were collected in EDTA (50 µM). The peritoneal washes were centrifuged at 1100 rpm for 5 minutes at RT and pelletted. Samples were resuspended for magnetic labeling in 80 µL of MACS® separation buffer (Miltenyi Biotec, Bergisch Gladbach, Germany). To remove epithelial cell component from the peritoneal wash and consequently to enrich the mesothelial portion, immunomagnetic depletion using anti-CD326/EpCAM microbeads (Miltenyi Biotec, Bergisch Gladbach, Germany) was performed according to the manufacturer’s instructions. Briefly, MS separation columns (MACS®, Miltenyi Biotec) had been equilibrated with 0,5 mL of MACS® separation buffer and the microbeads labeled cells were subjected to magnetic field trough the column passage. The CD326 negative cells were washed off from the column, and were plated in DMEM/F12 as above.

For the adhesion experiments, MeT-5A or HPMCs were grown to confluence and after 24h Caco2 or AGS cells were seeded on the monolayer.

### Morphological analysis of HPMCs

For the HPMCs morphological analysis, the culture samples were observed on a Zeiss Axiovert 200 inverted microscope equipped with phase contrast (DIC) optics (Zeiss, Oberkochen, Germany). Quantitative analysis of multinucleated and multivacuolated cells was performed by counting, for each cell culture, a total of at least 250 cells observed in five microscopic fields randomly taken from three different experiments. All results were expressed as mean values ± SE. Significance was calculated using Kruskal-Wallis test or paired Student’s t test. *P* values <0.05 were considered statistically significant.

### Immunofluorescence

For HPMCs characterization cells were grown on coverslips and fixed with 4% paraformaldehyde followed by treatment with 0,1 M glycine for 20 minutes at 25°C and with 0,1% Triton X100 for an additional 5 minutes at 25°C to allow permeabilization. Cells were then incubated for 1 hour at 25°C with the following primary antibodies: anti-cytokeratins (recognizing CK8 and CK19 among other CKs) (1∶100 in PBS; clone MNF116; Dako, Glostrup, Denmark) monoclonal antibody; anti-vimentin (1∶100 in PBS; clone V9; Dako) monoclonal antibody; anti-calretinin (1∶100 in PBS; clone DAK Calret 1; Thermo Fisher Scientific Inc., Fremont, CA, USA) monoclonal antibody; anti-CEA (1∶100 in PBS; Zymed, Invitrogen, Carlsbad, CA, USA) polyclonal antibodies; anti-EpCAM (1∶10 in PBS; Miltenyi Biotec GmbH, Bergisch Gladbach, Germany) monoclonal antibody directly conjugated with PE; anti-ICAM1 (1∶10 in PBS; Stemcell Technologies, Vancouver, BC, Canada) monoclonal antibody directly conjugated with FITC.

The unconjugated primary antibodies were visualized, after appropriate washing with PBS, using goat anti-mouse FITC (1∶50 in PBS; Cappel Research Products, Durham, NC), goat anti-mouse Texas Red (1∶200 in PBS; Jackson Immunoresearch Laboratories, West Grove, PA, USA), goat anti-rabbit FITC (1∶400 in PBS; Cappel Research). To identify cycling cells, immunostaining was performed with anti-Ki67 rabbit polyclonal antibodies (1∶50 in PBS; Zymed Laboratories, San Francisco, CA). Nuclei were stained with 4′,6-diamidino-2-phenylindole (DAPI) (1∶10.000 in PBS; Sigma). Coverslips were finally mounted with 90% glycerol in PBS for observation. Fluorescence signals were visualized with the ApoTome System (Zeiss) connected with an Axiovert 200 inverted microscope (Zeiss) and image analysis was performed by the Axiovision software (Zeiss) and KS300 Image analyzer software (Zeiss).

Percentage of EpCAM/Ki67-positive cells in co-cultures of MeT-5A and AGS or Caco2 cells was analyzed counting a total of 500 cells randomly observed in 5 microscopic fields for each different time points (1h, 24h, 48h) during the time course of the experiment. Percentage of ICAM1-positive cells in HPMCs was analyzed counting for each primary culture a total of 300 cells, randomly observed in 10 microscopic fields from three different experiments. Quantitative analysis of the ICAM1 fluorescence intensity was performed by the analysis of 100 cells for each sample in five different fields, randomly taken from three different experiments. All results were expressed as mean values ± SE. Significance was calculated using Kruskal-Wallis test or Student’s t test; *p* values < 0.05 were considered statistically significant.

### Adhesion assay

Subconfluent Caco2 or AGS cells were trypsinized and resuspended in DMEM serum free and labeled with 5 µl/ml of Vybrant®DiI solution (Invitrogen, Carlsbad, CA, USA) by incubation for 30 minutes at 37°C. The DiI-labeled cells were washed three times and resuspended in DMEM/F12 as above. The labeled-cells were directly plated on the mesothelial monolayer (25×10^3^/cm^2^ of monolayer) and incubated for 1, 24, 48 hours. In the adhesion assays with the anti-ICAM1 blocking antibody (Stemcell Technologies), the incubation was performed in the presence of different dilutions (1∶10, 1∶5 and 1∶2) of the antibody. An unrelated antibody (anti-cytokeratins; clone MNF116; Dako) was used as negative control at 1∶2 dilution. Non-adherent cells were removed by abundant washes with serum free medium, and adherent cells and HPMCs monolayers were fixed with 4% paraformaldehyde, followed by treatment with 0.1 M glycine for 20 minutes at 25°C and with 0.1% Triton X-100 for additional 5 minutes at 25°C to allow permeabilization. Nuclei were stained with DAPI. Nuclei were stained with DAPI (4′,6-diamidino-2-phenylindol) (1∶10.000 in PBS; Sigma). Quantitative analysis of DiI-positive cells/mm^2^ was performed by counting the number of positive cells in 10 different optical fields of 2,24 mm^2^, randomly taken from three different experiments. Results have been expressed as mean values ± SE. P values were calculated using Kruskal-Wallis test and significance level was defined as p<0.05.

### Reactive oxygen species detection

For reactive oxygen species (ROS) detection, HPMCs cells were incubated with 2′,7′-dichlorofluorescein diacetate (DCFH-DA, Fluka) (5 µM) for 10 min at 37°C, washed extensively with PBS and immediately observed under an Axioskop 2 microscope equipped with Pascal LSM 5 confocal laser scan (Zeiss, Oberkochen, Germany) using an argon laser with a 488 nm excitation band. The emission long pass was a 505 filter: laser intensity, pinhole diameter and photomultiplier settings were kept constant for every experiment [27]. For the optimization of the method, HPMCs #062 at passage 2 were treated with the pro-oxidant Cumene Hydroperoxide (Fluka Chemika, AG, Buchs, Switzerland) at different doses (100 and 200 µM) in presence or not of the anti-oxidant Vitamin E (15 µg/ml) before the addiction of DCFH-DA. The fluorescence intensity (FUI, Fluorescence Intensity Units) was measured by Zeiss KS300 image analyzer software (Zeiss) evaluating at least 200 cells for each condition in three different microscopic fields. The data presented are expressed as mean values ± SE from three different experiments. Statistical analysis was performed using paired Student’s t test and significance level has been defined as *p* <0.05.

The generation of basal intracellular ROS was also measured by cytofluorimetric assay. The #062 HPMCs, treated with DCFH-DA as above, were trypsinized, pelleted, resuspended in pre-warmed medium and collected with MACSQuant® Analyzer flow cytometer (Miltenyi Biotec GmbH). Excitation and emission wavelengths were 488 and 525 nm respectively (FL2 channel). The green fluorescence signal was analyzed by MACSQuantify® software (Miltenyi Biotec GmbH) and visualized on a three-decade log scale. The mean fluorescence intensity (MFI) was calculated from three independent experiments with evaluation of at least 20,000 events for assay and expressed as relative fluorescence intensity (mean±SE). Statistical analysis was performed using paired Student’s t test with significance level defined as *p* <0.05.

## Results

### Optimization of the in vitro test for evaluation of the adhesion of cancer cells to the mesothelial monolayers

**Figure 1:**
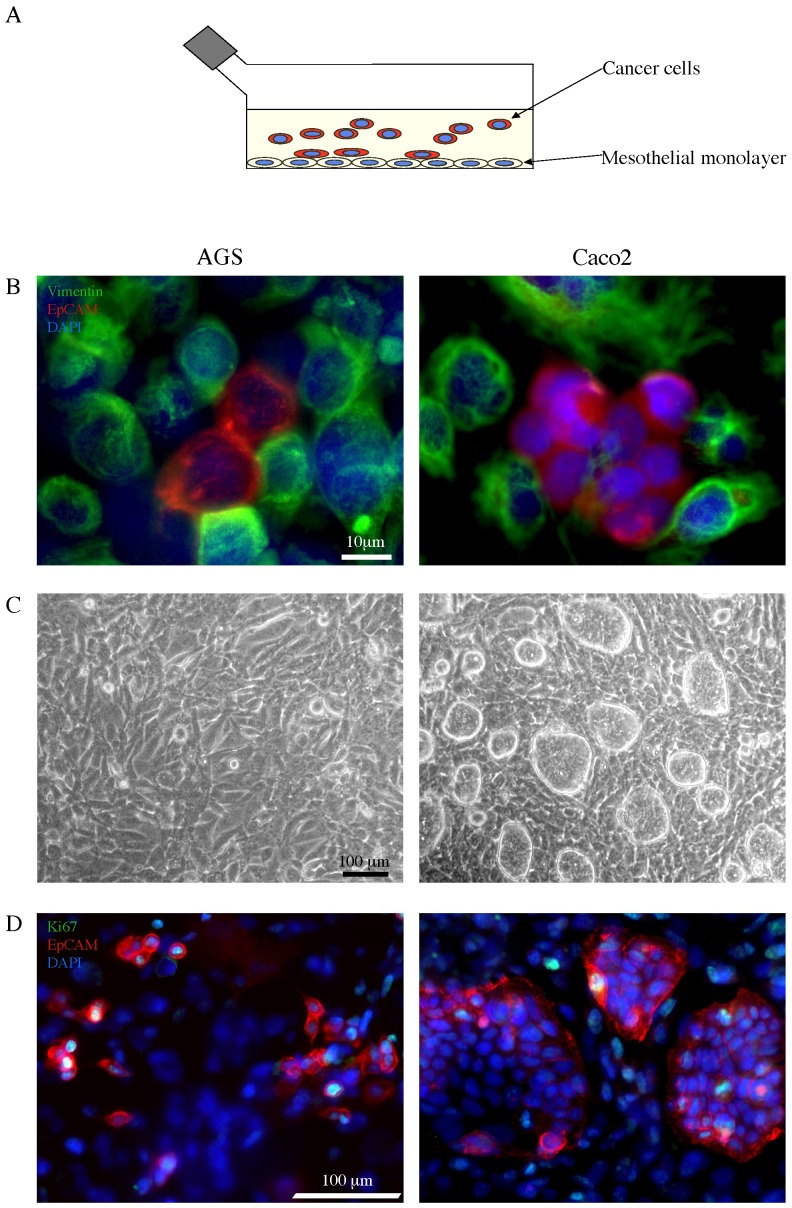
Co-culture *in vitro* test for the adhesion of cancer cell lines to mesothelial monolayer. A) Schematic drawing of the co-culture system and the adhesion test used throughout the study: cancer cells are seeded on a mesothelial monolayer to evaluate cell adhesion. B) MeT-5A mesothelial cell line was grown at confluence and AGS or Caco2 cells were seeded on the mesothelial monolayer in co-culture (25.000 cells/cm^2^). After 24 hours from seeding, the co-culture was fixed, permeabilized and stained with a primary antibody directed against vimentin, followed by a secondary Ab labeled with the FITC fluorocrome (green) to identify the mesothelial cells. Double immunofluorescence with α-EpCAM PE antibody (red) was performed to recognize the cancer epithelial cells. Cellular nuclei were stained with DAPI (blue). The immunofluorescence analysis reveals the different cell types in our co-culture model. The signal corresponding to vimentin in the cell monolayer is compatible with that of intermediate filaments, as perinuclear cytoplasmic bundles, while the EpCAM staining is associated with the plasma membrane of the cancer cells. Both AGS and Caco2 cells appear either in small clusters or isolated and strictly adherent to the mesothelial cells. Bar: 10 µm. C) Phase contrast microscopy used to verify the integrity of mesothelium monolayer. After 48 hours from seeding, the adherent Caco2 cells display a pattern of growing in compact islands, while the AGS adhering cells show a more flattened shape and an isolated pattern of growth. Bar: 100 µm. D) Proliferation assay performed by immunofluorescence staining with a primary anti-Ki67 antibody, which identifies cycling cells, followed by a secondary FITC-labeled Ab (green). The tumor cells were labeled with the anti-EpCAM PE Ab as above. After 48 hours from seeding, the distribution of the cancer cells positive for the Ki67 nuclear signal reveals a different behavior of tumor growth: differently from the isolated AGS cells, the Ki67^+^ Caco2 cells are located at the periphery of the islands. Bar: 100 µm.

One of the first key step in peritoneal metastatic dissemination of gastrointestinal tumours is the adhesion of cancer cells to the mesothelial monolayer [4]. To study the biological behaviour of both cancer and mesothelial cells and to evaluate their properties of adhesion, we first selected and adapted to our conditions a co-culture system and an in vitro test for adhesion (Fig. 1A), previously used for ovarian cancer [12]. The human mesothelial cell line MeT-5A was grown at confluence and human gastric adenocarcinoma cells (AGS cell line) or human colon carcinoma cells (Caco2 cell line) were seeded in co-culture at the density of 25.000 cells/cm^2^ of mesothelial monolayer.

To identify the different cell types in our co-culture model, we used immunofluorescence (IF) microscopy. After 24 hours from seeding, to recognize the mesothelial cells making up the Met-5A monolayer, we stained the co-cultures with a primary antibody directed against vimentin, a component of the intermediate filaments of the cytoskeleton, followed by a secondary Ab labeled with the FITC fluorocrome (green): the signal was compatible with the structure and localization of vimentin, which appears as perinuclear cytoplasmic bundles of filaments (Fig. 1B). The cancer cells were labeled with α-EpCAM PE antibody, recognizing a human epithelial adhesion molecule and directly conjugated to the fluorochrome PE (red): the corresponding signal was associated with the plasma membrane of the cells adherent to the monolayer (Fig. 1B). The cellular nuclei were stained with DAPI (blue). Both AGS and Caco2 cells appeared either in small clusters or isolated and strictly adherent to the mesothelial cells (Fig. 1B).

For the evaluation of the adhesive properties of the cancer cells, we used phase contrast microscopy, which allowed to verify the mesothelium monolayer and removed any doubt about the possibility of cancer cells adhering to the glass or plastic support. The morphological analysis after 48 hours from seeding showed that the adherent Caco2 cells displayed a pattern of growing in compact islands (Fig. 1C). In contrast, the AGS adhered cells were characterized by a more flattened shape and a more isolated pattern of growth (Fig. 1C).

To better understand the biological behaviour observed in phase contrast microscopy and to evaluate the proliferation rate of the adherent cells, we used IF analysis with the Ki67 marker which identifies cycling cells. After 48 hours from seeding, the co-cultures were stained with a primary anti-Ki67 antibody, followed by a secondary FITC-labeled Ab (green). The tumor cells were labeled with the anti-EpCAM PE Ab as above. While the proliferative rate of the two adhering cell types, evaluated as the percentage of the cells positive for the Ki67 nuclear signal was comparable (21% ±2 and 23% ±2 for the Caco2 and AGS cells respectively; Kruskal-Wallis test: *p*  = NS), their distribution revealed a different behaviour of the cancer cells (Fig. 1D). In fact, unlike AGS cells, the Ki67^+^ Caco2 cells were located at the periphery of the islands, as expected from their spontaneous ability to differentiate in vitro [26].

**Figure 2:**
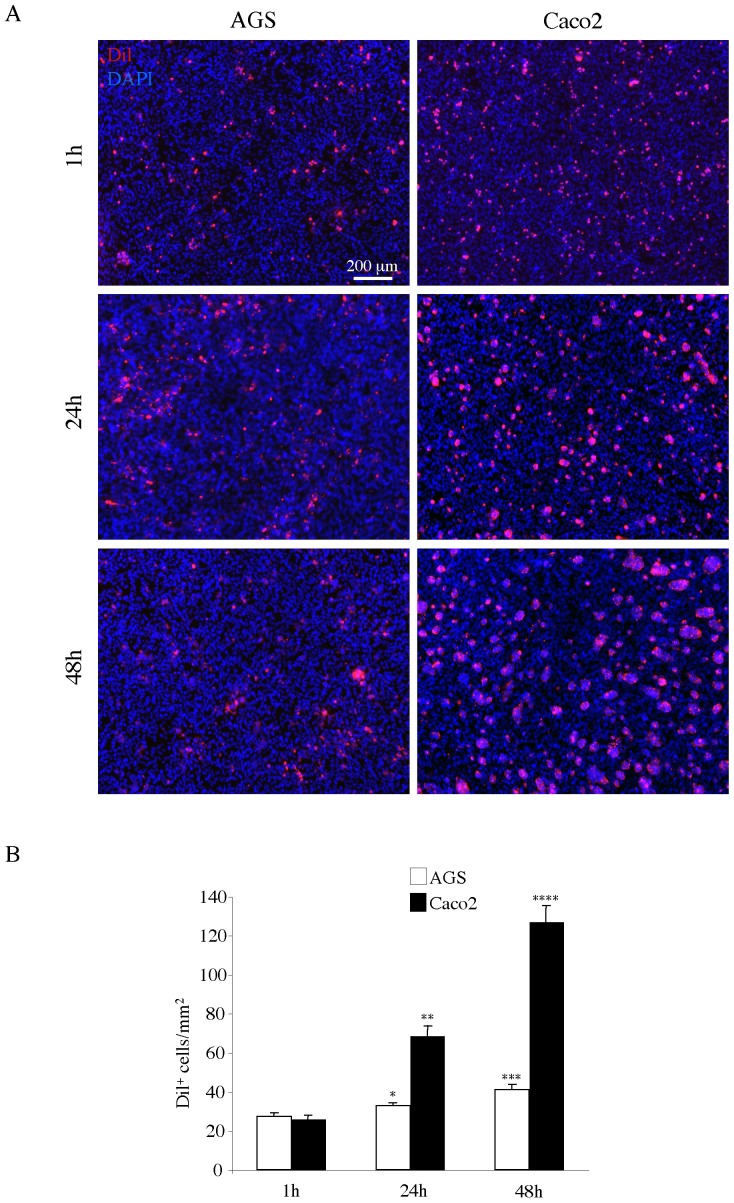
Adhesion test with AGS and Caco2 cells on Met-5A monolayer. A) Met-5A mesothelial monolayer was grown as described above. Caco2 and AGS cells were labeled with the DiI tracer and then seeded on the monolayer as above. After 1, 24 and 48 hours, co-cultures were washed, fixed and permeabilized. Nuclei were stained with DAPI. Bar: 200 µm. B) Quantitative analysis of the number of adherent DiI^+^ cells/mm^2^ was performed as described in materials and methods. While after 1 hour of seeding both Caco2 and AGS cells adhere to the monolayer in equal amount, at the 24 and 48 hours time points the number of Caco2 cells is almost doubled compared to that of AGS, which increases only slightly but significantly over the time. Results are expressed as mean values ± IC 95%. Statistics: Student’s t test: *p <0.01 vs AGS 1 hour; **p <0.001 vs Caco2 1 hour and AGS 24 hours; ***p <0.01 vs AGS 1 hour and *p*  = NS vs AGS 24 hours; ****p <0.001 vs Caco2 24 hours.

For a quantitative evaluation of the adhesion of the two cancer cell lines to the MeT-5A monolayer, we used the lipophilic cellular tracer DiI to label the cancer cells before the adhesion test [12]. Figure 2A shows the results obtained by the contemporary use of DiI and DAPI staining of the co-cultures at different time points (1h, 24h, 48h) from seeding. Images of 10 different optical fields were randomly taken as described in materials and methods. The numbers of DiI^+^ cancer cells per mm^2^ were then calculated and statistically analyzed as described in materials and methods. The results in figure 2B showed that both Caco2 and AGS cells were adhering to the mesothelial monolayer in equal amount at 1 h of co-culture. However, adhesion of Caco2 cells had the tendency to double after 24 and 48 hours, while the AGS cells, although slightly but significantly increasing in number during the timespan, were less numerous than the Caco2 cells at either time points (*p* <0.05). Because the proliferative rate of the two cell types at 48 hours, as described above, did not reveal differences which may account for the higher number of Caco2 cells adhering to the monolayer compared to the AGS cells, the results of the DiI-based test appeared to reflect real differing adhesive properties.

### Adhesion of cancer cells to primary human mesothelial monolayer derived from peritoneal washes

To assess the possible role of the mesothelium in the adhesion process of the cancer cells in our co-culture system, we used the above test with primary cultures of mesothelial cells obtained from the peritoneal wash of patients affected by peritoneal carcinomatosis from colorectal or gastric cancer and non-carcinoma disease. In fact, the peritoneal lavage represents a practical source of mesothelial cells [19], instead of utilizing omentum fragments.

**Figure 3:**
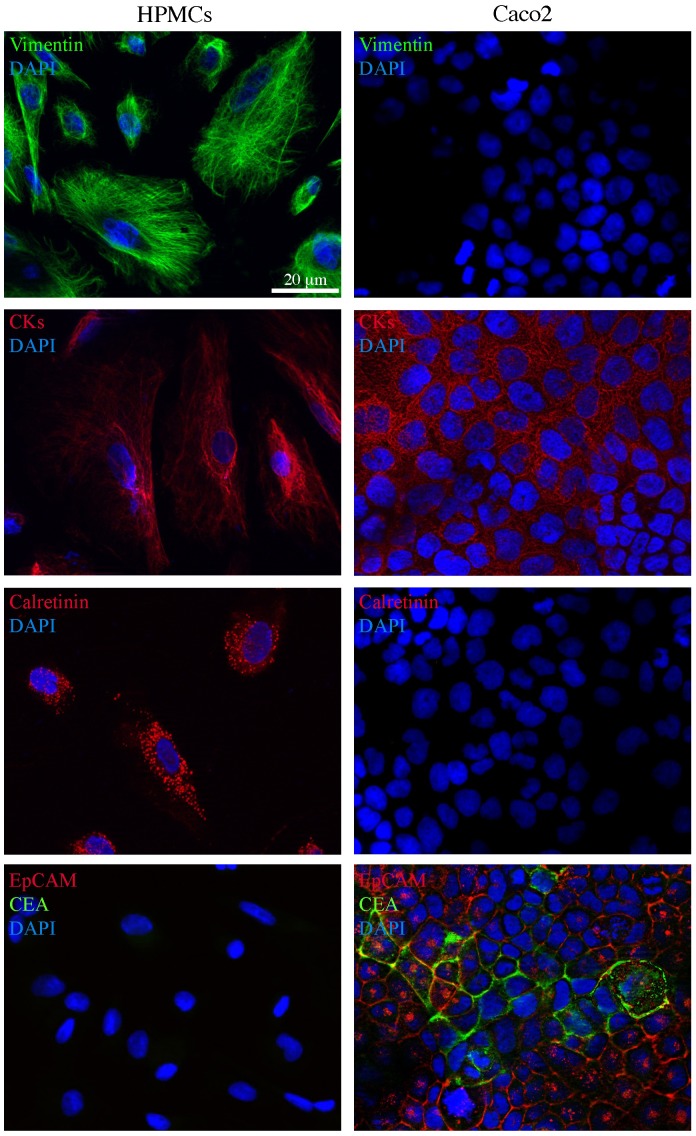
Immunofluorescence characterization of human peritoneal mesothelial cells from peritoneal washes of gastric and colon cancer patients. Primary cultures of human peritoneal mesothelial cells (HPMCs) were isolated from peritoneal washes as described in materials and methods. Caco2 colon cancer cells were used as a control. Immunofluorescence analysis using antibodies directed against mesothelial (vimentin, CK8 and CK19 cytokeratins and calretinin) and epithelial (EpCAM and CEA) markers shows that HPMCs are positive for vimentin and cytokeratin staining, that appears as perinuclear bundles of filaments, as well as for the hot-spotted calretinin signal, but are negative for the plasma membrane EpCAM staining and for the intracellular and surface CEA signal. Caco2 cells are positive for cytokeratins and double positive for the EpCAM and CEA epithelial markers visible on the cell surfaces (EpCAM, green signal) or on the plasma membranes and in intracellular spots (CEA, red signal). Nuclei were stained with DAPI. Bar: 20 µm.

To characterize the human peritoneal mesothelial cells (HPMCs), obtained as described in materials and methods, we used immunofluorescence microscopy (Fig. 3). To recognize the primary mesothelial cells from other types of cells present in the peritoneal wash, such as fibroblasts and epithelial cancer cells, we stained the cultures with a combination of antibodies directed against known mesothelial markers, such as vimentin, cytokeratins (CK8 and CK19) and calretinin. To be sure that the cells were of mesenchymal origin and not epithelial, we used in parallel the same antibodies on Caco2 cells. The results showed that the HPMCs were positive for both vimentin and cytokeratin staining, which appeared as perinuclear cytoplasmic bundles of intermediate filaments (Fig. 3, left panels). As expected, Caco2 cells were negatively stained for vimentin and positively labeled for cytokeratins (Fig. 3, right panels). To unequivocally discriminate the HPMCs from fibroblasts possibly present in our cultures, cells were labeled with antibodies against calretinin, an intracellular calcium-binding protein belonging to the troponin-C superfamily expressed in mesothelial cells: the signal was in cytosolic hot-spots (Fig. 3, right panel). Again, the epithelial Caco2 cells were negative (Fig. 3, left panel). In contrast, HPMCs were negative for the epithelial marker EpCAM which was expressed on the plasma membranes of the Caco2 cells (Fig. 3, bottom panels, red signal) and for the tumor marker carcinoembryonic antigen CEA, whose signal was visible either in intracellular spots or on the cell surfaces (Fig. 3, bottom panels, green signal).

**Figure 4:**
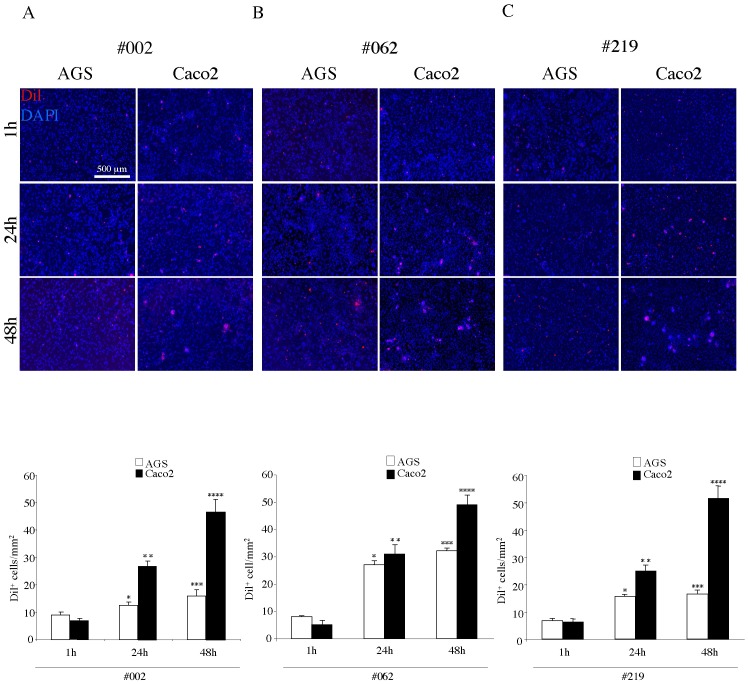
Adhesion test with AGS and Caco2 cells on different HPMC monolayers. HPMCs isolated from the peritoneal wash of a non-cancer patient (A, #002), from that of a colon cancer patient (B, #062) and from that of a gastric cancer patient (C, #219), were grown to confluent monolayer as above. Caco2 and AGS cells were labeled with DiI, seeded on the HPMC layers, left to adhere for different time points (1, 24 and 48 hours) and then washed, fixed and permeabilized. Nuclei were stained with DAPI. Quantitative analysis of the number of adherent DiI^+^ cells/mm^2^ was performed as described in materials and methods. Independently on the origin of the peritoneal washes, the Caco2 cells show higher levels of adhesion respect to AGS at 24 and 48 hours. However, while the adhesion of the Caco2 cells is similar to all mesothelial layers, the AGS cells display significant differences, showing higher adhesion to the layer #062 respect to the #219 and the #002. Results of the quantitative analysis are expressed as mean values ± IC 95%. Kruskal-Wallis test: A) *p<0.05 vs the AGS 1 hour; **p <0.01 vs Caco2 1 hour, *p* <0.01 vs AGS 24 hours; ***p <0.05 vs the AGS 1 hour and *p*  = NS vs the AGS 24 hours; ****p <0.01 vs Caco2 24 hours. B) *p <0.01 vs the AGS 1 hour; **p <0.01 vs Caco2 1 hour, *p*  = NS vs AGS 24 hours; ***p <0.01 vs the AGS 1 hour, *p*  = NS AGS 24 hours; ****p <0.01 vs Caco2 24 hours. C) *p <0.01 vs the AGS 1 hour; **p <0.01 vs Caco2 1 hour, *p* <0.01 vs AGS 24 hours; ***p <0.05 vs the AGS 1 hour, *p*  = NS 24 AGS hours; ****p <0.001 vs Caco2 24 hours.

For a quantitative evaluation of the ability of the two cancer cell lines (AGS and Caco2 cells) to adhere to different HPMC monolayers, we used the DiI tracer as above to mark the cancer cells before the adhesion test. For the analysis we utilized three primary cultures of mesothelial cells, derived from the peritoneal washes of patients without carcinoma disease (Fig. 4A), with colorectal cancer (Fig. 4B) or with gastric cancer (Fig. 4C) and we were able to compare the contribution of different mesothelial monolayers to the adhesion of the same type of cancer cells at different time points (1h, 24h, 48h). The quantitative analysis of the adhesion of DiI^+^ cells to the HPMC monolayers (Fig. 4A–C) showed reduced levels of adhesion in timespan for both tumor cell lines compared to the adhesion test performed on MeT-5A (see Fig. 2B). On these primary cultured monolayers, the Caco2 cells were more adherent than AGS cells at either 24 or 48 hours, independently on the origin of the peritoneal washes. However, while the adhesion of the Caco2 cells was comparable to all mesothelial layers, irrespectively on their source from patients with neoplastic or benign disease, the AGS cells display significant differences in their behaviour, showing higher adhesion to the HPMCs from colon cancer patient (#062) respect to the HPMCs from either gastric cancer patient (#219) or from non-carcinoma disease (#002). Thus, while the adhesion properties of the mesothelial monolayers appear independent on the cancer environment, our co-culture model is able to detect differences among the HPMCs.

### Role of HPMC senescence in the adhesion process

**Figure 5:**
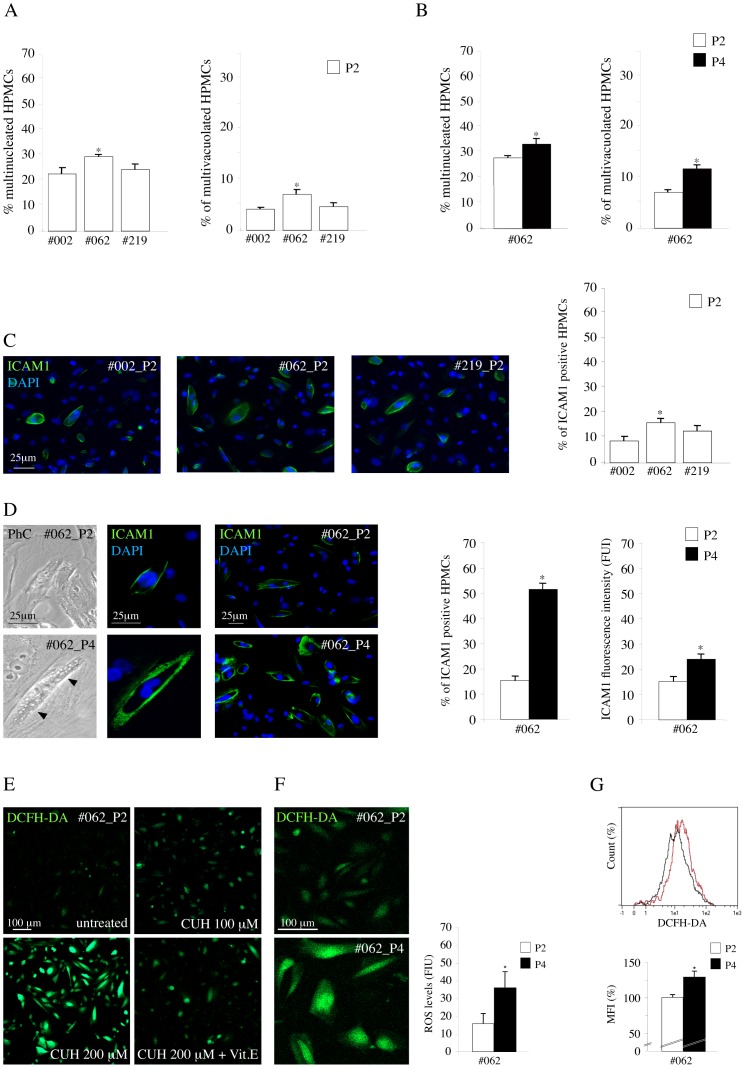
Morphological features, expression of ICAM1 and intracellular ROS production in HPMC monolayers during in vitro induced senescence. A) Quantitative evaluation of senescence-related morphological features in HPMC monolayers from the peritoneal wash of the non-cancer patient (#002), from that of the colon cancer patient (#062) and from that of the gastric cancer patient (#219) after the first confluence (P2). The #062 HPMCs shows an higher percentage of multinucleated and multivacuolated cells respect to the other mesothelial cells (Kruskal-Wallis test: *p <0.05 vs #002 and #219). B) Quantitative evaluation of the senescence morphological features in #062 HPMC monolayer during in vitro induced senescence. The percentages of multinucleated and multivacuolated cells increases from passage 2 (P2) to passage 4 (P4) confirming the enhanced level of senescence of P4 (paired Student’s t test: *p <0.05). C) Quantitative evaluation of the percentage of ICAM1 positive cells in the HPMC monolayers. The HPMCs #062 from the colon cancer patient shows an higher percentage of ICAM1 positive cells respect to the other mesothelial monolayers (Kruskal-Wallis test: *p <0.01 vs #002 and *p* <0.05 vs #219). D) Immunofluorescence analysis of the percentage of ICAM1 positive cells in #062 HPMC monolayer during in vitro induced senescence. The quantitative analysis of the percentage of ICAM1 positive cells in P2 and P4 passages shows the increase in the percentage of positive cells from P2 to P4. Quantitative immunofluorescence analysis with anti-ICAM1 antibodies shows that both the number of ICAM1 positive cells, displaying a clear plasma membrane staining, and the fluorescence intensity of the signal are increased in P4 cultures respect to P2 HPMCs. The parallel phase contrast observations show that the ICAM1 positive cells are enlarged, multinucleated and vacuolated as expected for senescent cells (left panel, arrowheads). The cellular nuclei were stained with DAPI. Results in the first graph are expressed as mean values ± SE; paired Student’s t test: *p<0.001 vs P2. The quantitative evaluation of the fluorescence intensity of the ICAM1 signal was performed as described in materials and methods: results in the second graph are expressed as mean values ± SE. paired Student’s t test: *p <0.01 vs P2. E) Optimization of the in vitro test for evaluation of ROS production in HPMCs from #062 patient. Treatment of HPMCs at P2 passage was performed with the pro-oxidant Cumene hydroperoxide (CUH) at different doses (100 and 200 µM) in presence or not of the anti-oxidant Vitamin E (15 µg/ml) before the addition of DCFH-DA. Increase of the fluorescence intensity is evident in cells treated with the oxidant in a dose-dependent manner, while a clear decrease in the signal is induced by the incubation with the anti-oxidant. F) Evaluation of ROS production in HPMCs #062 at P2 and P4 passages was performed with addition of DCFH-DA (2′,7′-dichlorofluorescein diacetate) and fluorescence detection by confocal microscopy as described in materials and methods. The increase in the fluorescence intensity signal of DCFH-DA in the late passage P4 compared with the earlier P2 confirm the enhancement of ROS generation induced by senescence of the mesothelial cells. Results are expressed as FUI mean values ± SE. Paired Student’s t test: *p <0.001 vs P2. G) Basal intracellular ROS generation in HPMCs #062 at P2 and P4 passages performed by cytofluorimetric assessment of DCFH-DA fluorescent signal. A representative flow cytometry histogram showed two distinct peaks of fluorescence intensity, with enhancement of DCFH-DA signal in HPMCs #062 P4 (purple histogram) respect to P2 (black histogram). The mean fluorescence intensity (MFI), calculated from three independent experiments, was higher in the late passage P4 compared with the earlier P2. Results are expressed as MFI relative values ± SE. Kruskal-Wallis test: *p <0.05.

To analyze by our model the contribution of possible cellular and molecular mechanisms which may play a role in the different adhesive properties of the HPMCs, we focused our attention on the mesothelial senescence. In fact, among the physiological characteristics of the mesothelial monolayer, the senescence level of HPMCs is believed to promote the adhesion of tumour cells [22]–[24]. Interestingly, our HPMCs, being derived from peritoneal washes instead of from omentum samples, displayed already at the first in vitro passage the well known features of senescence, like an enlarged morphology, multiple nuclei and cytoplasmic vacuolization [14]. The quantitative analysis of these senescence-related morphological findings was performed in the three primary cultures used above and showed that the percentages of multinucleated and multivacuolated cells were higher in HPMCs from the colon cancer patient (#062) respect to the other HPMCs (Fig. 5A). In addition, since in the study of Ksiazek et al. [23] senescence of human omentum-derived peritoneal mesothelial cells was induced in vitro to analyze its effect on tumour cell adhesion, we applied a similar approach inducing the senescence of our primary cultures by sequential passaging.

To this aim, we compared the #062 primary culture of HPMCs from colon cancer patient, which appeared to be the more senescent, at two different passages: P2, obtained by seeding after the first confluence as above, and P4, after 2 passages 1∶3 from P2, as reported [23]. The morphological quantitative analysis showed the increased percentages of multinucleated and multivacuolated cells (Fig. 5B) from passage 2 (P2) to passage 4 (P4) confirming the enhanced level of senescence of P4 (paired Student’s t test: *p <0.05).

Because it has been proposed that the peritoneal senescence correlates with an increase of the expression of the intercellular adhesion molecule 1 (ICAM1) on the plasma membrane as a consequence of the oxidative stress [24], we wondered if we could observe differences in ICAM1 expression in our selected HPMCs. To this purpose, we evaluated by quantitative immunofluorescence the percentage of ICAM1 positive cells in HPMC monolayers after the first confluence: consistent with the increased senescent state, the HPMCs from colon cancer patient (#062), which appeared also to better contribute to the adhesion of the cancer cells in the experiments previously described (Fig. 4), showed an higher percentage of ICAM1 positive cells respect to the other mesothelial cells (Fig. 5C). To further correlate the ICAM1 expression with the senescent behaviour, we induced the in vitro senescence by sequential passaging, as above. The phase contrast microscopic analysis showed an increase in the cell size and in the number of vacuolated cells (Fig. 5D arrowheads), reflecting the increase in the level of senescence from P2 to P4. In addition, because peritoneal senescence correlates with an increase of the expression of the intercellular adhesion molecule 1 on the plasma membrane as a consequence of the oxidative stress [24], we confirmed the induction of senescence in our cultures by quantitative immunofluorescence with anti-ICAM1 antibodies (Fig. 5D): the results demonstrated that either the percentage of ICAM1-positive cells or the fluorescence intensity of the ICAM1 signal on the cell surface, assessed as described in materials and methods, were clearly increased from passage P2 to P4. The ICAM1-positive cells were larger than the negative cells in the same culture and frequently appeared multinucleated and vacuolated as observed also in the corresponding phase contrast images (Fig. 5D), further demonstrating that HPMCs at P4 were more senescent respect to P2.

For an additional assessment of senescence-related features, we investigated the oxidative state of our P2 and P4 cultures evaluating the basal intracellular production of reactive oxygen species (ROS). To this purpose, we performed addition of DCFH-DA (2′,7′-dichlorofluorescein diacetate) and fluorescence detection by confocal microscopy and flow cytometry. Figure 5E showed the results of the preliminary experiments to set up the assay for ROS detection by confocal microscopy, as described in materials and methods. A dose-dependent increase in fluorescence intensity was observed when cells were treated with the pro-oxidant Cumene Hydroperoxide, while a clear decrease of ROS levels was induced by the presence of the anti-oxidant Vitamin E, demonstrating the reliability of the assay. Then, we compared the levels of ROS production in P2 and P4 cultures: as shown in figure 5F, the results obtained by the quantitative fluorescence analysis were consistent with an increase of fluorescent cells in the P4 late passage compared with the P2 early one, in agreement with the literature [23]. These findings were further confirmed by cytofluorimetric assessment of DCFH-DA fluorescent signal (Fig. 5G).

**Figure 6:**
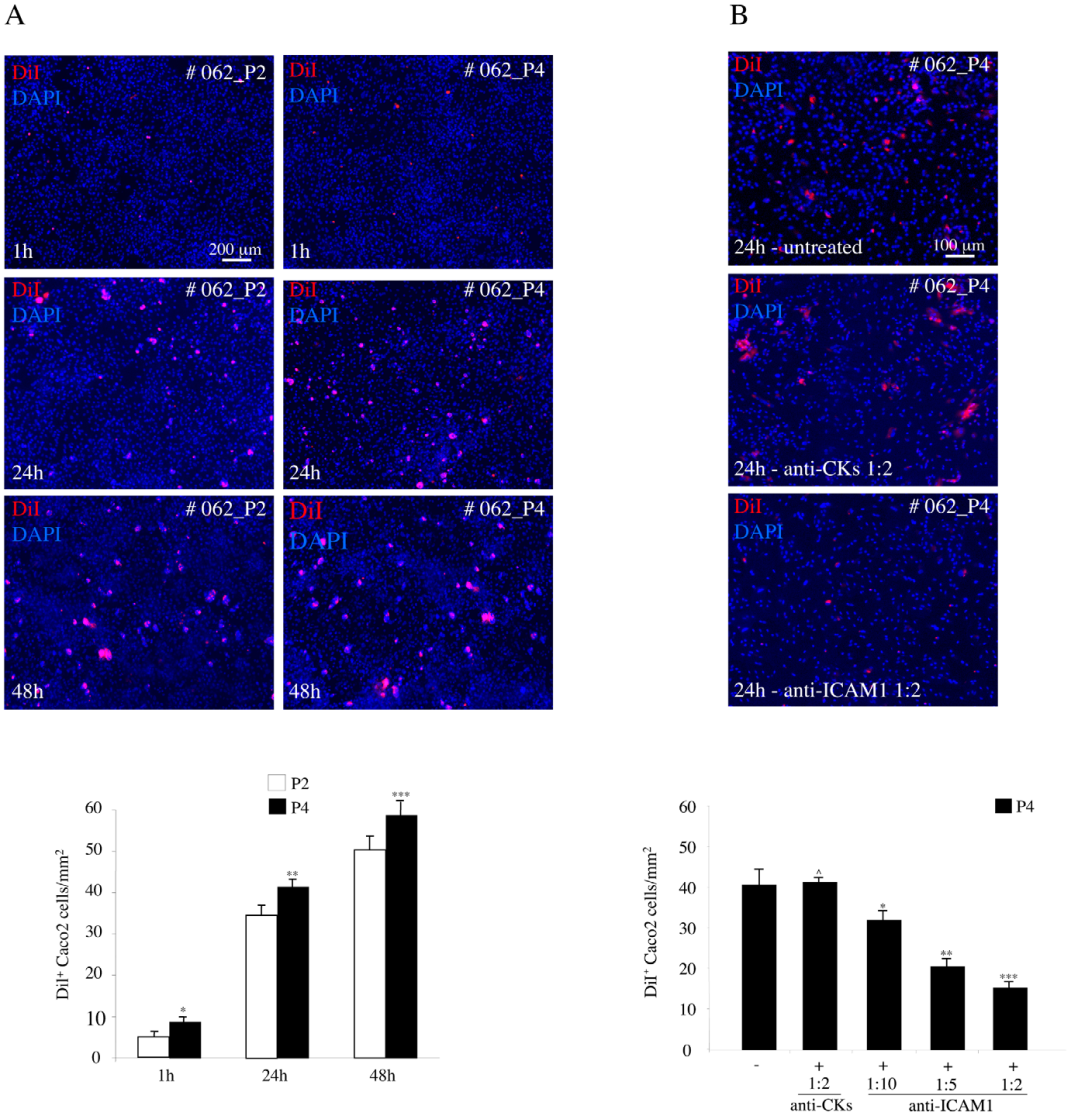
Adhesion test with Caco2 cells on senescent HPMC monolayer. A) HPMCs from the #062 peritoneal wash were cultured at P2 and P4 as described in figure 5. Caco2 cells were labeled with DiI, seeded on the HPMC layers, left to adhere for different time points (1, 24 and 48 hours) and then washed, fixed and permeabilized. Nuclei were stained with DAPI. B, C) Quantitative analysis of the number of adherent DiI^+^ cells/mm^2^ was performed as described in materials and methods. In B, the number of cancer cells adhering to the HPMC monolayer at P4 is significantly increased respect to the values in P2 at all time points. In C, the addition of decreasing dilutions of an anti-ICAM1 blocking antibody at the representative 24 hours time point leads to a progressive dose-dependent inhibition of the cancer cell adhesion to HPMCs at P4, while the addition of an unrelated antibody has no blocking effects on cancer cell adhesion. Results in B are expressed as mean values ± SE. Kruskal-Wallis test: *p <0.05 vs the P2 at 1 hour; **p <0.05 vs the P2 at 24 hours**;** ***p <0.05 vs the P2 at 48 hours. Results in C are expressed as mean values ± SE. Kruskal-Wallis test: ˆp <0.05 vs the absence of blocking antibody; *p <0.05 vs the absence of blocking antibody; **p <0.01 vs the antibody dilution 1∶10**;** ***p <0.05 vs the antibody dilution 1∶5.

To determine if the different levels of senescence could affect the adhesion of the cancer cells to the mesothelial monolayers, we evaluated, through the in vitro test used above, the ability of the Caco2 cells to adhere to the cultures of HPMCs at the different passages, P2 and P4. The results obtained by the contemporary use of DiI and DAPI staining of the co-cultures at various time points (1h, 24h, 48h) from seeding, showed a significant increase in the number of cancer cells adhering to the late P4 respect to the early P2 passages (Fig. 6A and 6B). To ascertain the possible involvement of the enhanced ICAM1 expression of the senescent cells in increasing the adhesion, we added decreasing dilutions of an anti-ICAM1 blocking antibody during the time course of the adhesion test: the antibody addition led to a progressive dose-dependent inhibition of the cancer cell adhesion (Fig. 6C), revealing that the ability of the cancer cells to better interact with senescent HPMCs is related to the increased expression of ICAM1 on the cell plasma membranes of the mesothelial cells, as reported [10], [24].

## Discussion

The role of the mesothelial cells in the process of cancer spreading in the peritoneal cavity has been, up to now, underestimated and remain to be clarified. However, similarly to the emerging crucial contribution of the stromal microenvironment surrounding the tumor tissue in the neoplastic progression, also the peritoneal layer is expected to represent a key mediator in the development of the carcinomatosis. The molecular mechanisms which may affect the interaction of the epithelial cancer cells to the mesothelium are probably quite analogous to those controlling the tumor cell adhesion to the endothelial layer during metastatic dissemination: both the fibrinolytic activity and the pattern of expression in adhesion molecules on the mesothelial or endothelial cells are major players in the process [4], [19]. In this paper, with the aim to investigate how the behaviour of mesothelial cells may differ depending on the tumor context of their origin as well as the possible state of activation or senescence, we propagated in vitro HPMCs isolated from different peritoneal washes of patients affected by colon or gastric cancers or from patients with benign diseases: in fact, the isolation of the mesothelial cells from the lavages, instead of from omental fragments, permits to obtain primary cultures resembling more closely the in vivo conditions, as suggested [19]. Consistent with what has been previously reported [14], we found that our primary cultures displayed all the morphological features and the marker positivity (vimentin, CK8, CK19 and calretinin) characteristic of the mesothelial cells.

For the adhesion test, we selected and optimized a co-culture method, previously proposed for ovarian cancer cells [12], based on the quantitative analysis of the adhesion of DiI^+^ cells to the HPMCs. First we set up the test using the mesothelial cell line MeT-5A and, when we moved to the primary cultures, we found reduced levels of adhesion of the cancer cells at all time points compared to the adhesion obtained with the MeT-5A monolayer, in agreement with the observations reported by Heyman et al. [12] utilizing the same DiI-based test. Our results with the HPMC layers, showing that both the AGS gastric carcinoma cells and the Caco2 colon carcinoma cells did not change their adhesion and growth when seeded on different mesothelial monolayers, indicated that the adhesive behaviour of the cancer cells was not affected by the origin and possible activation state of the HPMCs associated with different cancers.

To demonstrate that our cultures of HPMCs from peritoneal washes would represent a more reliable model of adhesion respect to other previously proposed with HPMCs from other sources, we first analyzed their expression of ICAM1, since this adhesion molecule is known to be more elevated in HPMCs from peritoneal wash comparing with cells from omental biopsies [15] and it has been recently reported that the increase in ICAM1 expression promotes the adhesion of cancer cells [10], [24]. In agreement with the reported observations [15], our primary cells showed an high expression of ICAM1 at early passages of the culture, suggesting that these detached cells present in the peritoneal fluid in vivo may possess adhesive properties more pronounced respect to the peritoneal intact layer. Interestingly, the HPMCs from peritoneal washes analyzed in our study were characterized also by the typical features of senescence already at the first in vitro P2 passages and by quite high levels of basal ROS production. Further increase of these features, i.e. ICAM1 expression and ROS generation, were obtained inducing in vitro senescence, as expected [22]–[24]. These acquired senescent state led to an increase in the adhesion of the cancer cells, which was inhibited by the addition of serial dilutions of a blocking anti-ICAM1 antibody, strengthening the role of ICAM1 in the adhesion process and suggesting that this ICAM1-mediated molecular interaction might be even more crucial for cells floating in the peritoneal fluid from which our cultures are derived.

## Conclusions

We suggest that the cancer environment might be not crucial for the peritoneal dissemination. However, we propose that the use of HPMCs from peritoneal washes would provide a practical and reliable tool for the in vitro analysis of the mesothelial molecular pathways involved in the adhesion process, the evaluation of the mesothelial conditions in cancer patients and the selection or validation of possible therapeutic strategies.
